# Superelastic Fire‐Safe Aerogel with Hierarchical Structures via Dual Templates Aided by Microbubble Engineering

**DOI:** 10.1002/advs.202506808

**Published:** 2025-06-19

**Authors:** Xiaoyang Yu, Huan Li, Ning Kang, Shouxiang Lu, Mingjun Xu, Man Pun Wan

**Affiliations:** ^1^ State Key Laboratory of Fire Science University of Science and Technology of China Hefei 230026 China; ^2^ School of Mechanical and Aerospace Engineering Nanyang Technological University Singapore 639798 Singapore

**Keywords:** aerogel, bubble engineering, gelatine, hierarchical structure, hyperelasticity

## Abstract

Superelastic aerogels with ultralow thermal conductivity have essential advantages for advanced thermal management systems in energy‐efficient buildings. However, inorganic aerogels suffer from brittleness and poor processability, whereas their organic counterparts experience high production costs and inadequate elastic recovery. This study used a dual‐template (ice and bubble) strategy to fabricate ultralight, superelastic aerogels with hierarchical porosity inspired by stress‐dissipating dome architectures. Microbubbles are engineered via a modified “Tessari method” to create macropores (≈100 µm) while ice‐templating introduced aligned pores of a few µm in size during freeze‐drying. The synergistic interplay of a rigid gelatine (Ge) skeleton, flexible polyvinyl alcohol (PVA) nodes, and potassium salt‐enhanced crystalline domains yielded aerogels with exceptional elasticity, ultralow density and thermal conductivity. Flame retardancy is achieved through potassium salt‐mediated catalytic carbonization, reducing the peak heat release rate by 54% and enabling self‐extinguishing behavior. Microbubble introduction in precursors can provide macropores for aerogels, which dispersed internal stress during the deformation of aerogel, whereas dynamic hydrogen bonds enabled rapid water‐assisted self‐healing ability and closed‐loop recyclability. Scalable production using commercial compressed air foaming systems and a low raw material cost further highlight its industrial viability. Combined with biodegradability and superior thermal insulation, this work advances sustainable, fire‐safe aerogels for multifunctional applications.

## Graphical abstract

Here, a dual‐template strategy (ice and bubble template) is used to prepare fire‐safe aerogels with hierarchical structures. When the foam expansion ratio exceeds the jamming transition value in the foam system, the aerogels demonstrate superelasticity and extreme shape adaptability. The as‐obtained aerogels can also be efficiently produced through water‐welding methods and demonstrate outstanding biodegradability and recyclability.

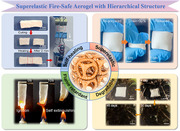

## 1 Introduction

Aerogels have demonstrated versatile applications in fields such as sensing, thermal insulation, and flame retardants owing to their unique characteristics of low density, high porosity, and exceptional thermal insulation properties.^[^ ^1^, ^2^, ^3^, ^4^, ^5^ ^]^ While classical inorganic silica aerogels have achieved large‐scale commercialization, their practical utilization is fundamentally constrained by the inherent brittleness of their silica networks.^[^ ^2^ ^]^ Moreover, petroleum‐derived precursors for silica aerogel production raise environmental concerns regarding sustainability. Organic polymer aerogels, including polyimide and syndiotactic polystyrene aerogels,^[^ ^6^, ^7^ ^]^ have emerged as candidates for thermal insulation textiles. However, these materials are plagued by a reliance on nonrenewable resources and prohibitive manufacturing costs. In contrast, renewable biomass‐derived aerogels based on nanocellulose and proteins have been developed for thermal management applications.^[^ ^8^, ^9^, ^10^ ^]^ Nevertheless, the industrial adoption of nanocellulose aerogels faces two critical challenges: i) Energy‐intensive extraction processes involving chemical treatments (e.g., TEMPO‐mediated oxidation) result in substantially elevated raw material costs (USD 30–100/kg^[^ ^11^ ^]^); ii) Most nanocellulose architectures exhibit inadequate elastic recovery (manifesting permanentcollapse after cyclic compression deformation exceeds 30%) due to irreversible hydrogen bonding between adjacent cellulose nanofibrils under compressive stress.^[^ ^12^ ^]^ This mechanical deficiency becomes particularly detrimental in aerogel applications in which insufficient resilience results in structural flattening under loading, ultimately causing pore collapse and consequent degradation of the thermal insulation performance.

In previous works, significant efforts have been devoted to constructing elastic aerogels, with structural unit geometry design emerging as a primary strategy for improving the mechanical properties of lightweight materials. A representative study revealed that interconnected lamellar architectures fabricated through bidirectional freeze‐casting enable graphene aerogels to withstand substantial deformation (≈80% strain) without plastic deformation or brittle collapse during compression.^[^ ^13^ ^]^ Zhang et al. developed elastic aerogels with fibrous porous networks through an electrospinning technique, leveraging dual diffusion mechanisms involving high‐volatility solvents and water vapor to achieve rapid non‐solvent‐induced phase separation.^[^ ^14^ ^]^ Furthermore, hierarchical structural design across multiple scales has proven to be effective in improving the elastic performance of porous aerogels, although the formation of these multiscale architectures typically requires complex manufacturing methods. For example, Xia et al. transformed thick cell walls in honeycomb‐structured graphene aerogels into pores with nanoscale walls. These ultralight nanostructured walls facilitate substantial elastic buckling deformation along the out‐of‐plane direction, thereby increasing mechanical resilience.^[^ ^15^ ^]^ Qin et al. employed a synergistic strategy that combines unidirectional freeze‐casting with thermal etching, in which ball‐milled cellulose nanofibres containing high‐aspect‐ratio PHA microparticles were homogenized into stable suspensions and then directionally frozen on a cryogenic copper platform to form hierarchically elastic porous aerogels.^[^ ^9^ ^]^ A dual ice‐templating approach has been developed for fabricating elastic cellulose aerogels, but the methodology necessitates a six‐step procedure involving two cycles of freezing/lyophilization, intermediate redispersion, and finally hydrophobic modification.^[^ ^16^ ^]^ The complexity of the above‐mentioned approach underscores the critical need for developing simplified fabrication routes to produce cost‐effective, hyperelastic biomass aerogels with multiscale architectures for practical applications.

The dual‐template (ice and bubble) strategy has recently become a promising approach for the fabrication of superelastic aerogels with hierarchical structures. The process used by Barbetta et al. to prepare aerogels through a foaming strategy involved high‐energy ball milling, initial foaming, cross‐linking, and freeze‐drying to degrade polysaccharides, successfully preparing elastic aerogels for cardiac tissue engineering.^[^ ^1^, ^3^ ^]^ Barbetta et al. used polysaccharides as aerogel skeletons, which involve environmentally unfriendly cross‐linking agents (to improve foam stability), as well as two rounds of freeze‐drying, foaming, and multi‐step purification processes.^[^ ^1^, ^3^ ^]^ Wang and Song et al. recently utilized aqueous polyurethane and cellulose nanofibres to generate foam, and after freeze‐drying, they prepared superelastic aerogels for clothing fillers and first proposed the “dome structure” concept combined with finite element simulation to explain the superelasticity of aerogels containing macropores.^[^ ^8^ ^]^ These studies show that bubble introduction in precursors can provide macropores for aerogels, which not only reduce wall rigidity and viscosity but also act as defect sites to guide microscopic deformation and disperse internal stress during macroscopic deformation.^[^ ^1^, ^3^, ^8^ ^]^ Other scholars noted that the introduction of macropores into aerogel precursors usually reduces the mechanical properties and elasticity of aerogels.^[^ ^17^ ^]^ For example, when the average pore size increased fourfold, the mechanical strength decreased by more than one‐third. Considering that the structure determines properties, aerogel elasticity may be affected by the foam bubble structure, which is influenced by the foam expansion ratio (ER). The formation of dome structures appears crucial for aerogel elasticity; however, until now, the critical ER value for dome structure formation in dual‐template‐fabricated superelastic aerogels has remained ambiguous.

In addition to controlling the foam ER, the inherent thermodynamic instability of foam is also critical for postfreezing‐drying aerogel structures. The main source of this instability lies in the large density difference between the gas phase and the liquid phase. Under a gravitational field, the liquid phase tends to drain downward, whereas gas rises upward. This causes simultaneous thinning and thickening of the liquid films surrounding the bubbles in the upper and lower foam regions. In extreme cases, a liquid layer forms at the foam bottom, whereas extensive coalescence occurs at the foam top. Therefore, strategies must be conceived to maximally restrict liquid‐phase drainage in foam templates.

The purpose of this study was to use low‐cost raw materials and a dual‐template strategy (ice template and bubble template) to prepare ultralight, superelastic aerogels with hierarchical structures, and to explore the formation conditions of dome structures that cause the superelasticity of aerogels. By incorporating Ge and PVA matrix with flame‐retardant nucleophilic potassium salts that improve the mechanical properties and inhibit liquid drainage through crystalline domain formation, we achieved structural control via microbubble formation engineering. Specifically, micron‐sized jammed bubble templates were created by pneumatically mixing air with Ge/PVA/potassium salt solutions, followed by pre‐freezing to establish ice templates. Subsequent freeze‐drying yielded aerogels with hierarchical porosity, where aligned pores of a few µm in size were generated on dome‐shaped macropore walls. This multiscale architecture enables exceptional deformation tolerance by effectively mitigating local strain concentrations. Within specific ER ranges, the formation of dome‐like structures and the superelasticity of aerogels were observed. When the ER of the foam exceeds the jamming transition value in the foam system, the obtained aerogels demonstrate superelasticity and extreme shape adaptability (foldable/twistable). Moreover, this biomass aerogel, which can be efficiently produced on a large scale through a water‐welding method using compressed air foam systems, demonstrates outstanding reparability and recyclability. Sustainable biomass aerogels also exhibit high performance in terms of thermal insulation and flame retardancy, further highlighting the advantages of hierarchical microstructures and excellent structural stability.

## 2 Results and Discussion

### 2.1 Design Strategy of Biomass Aerogels with Hierarchical Structures via the Tessari Method

**Figure 1:**
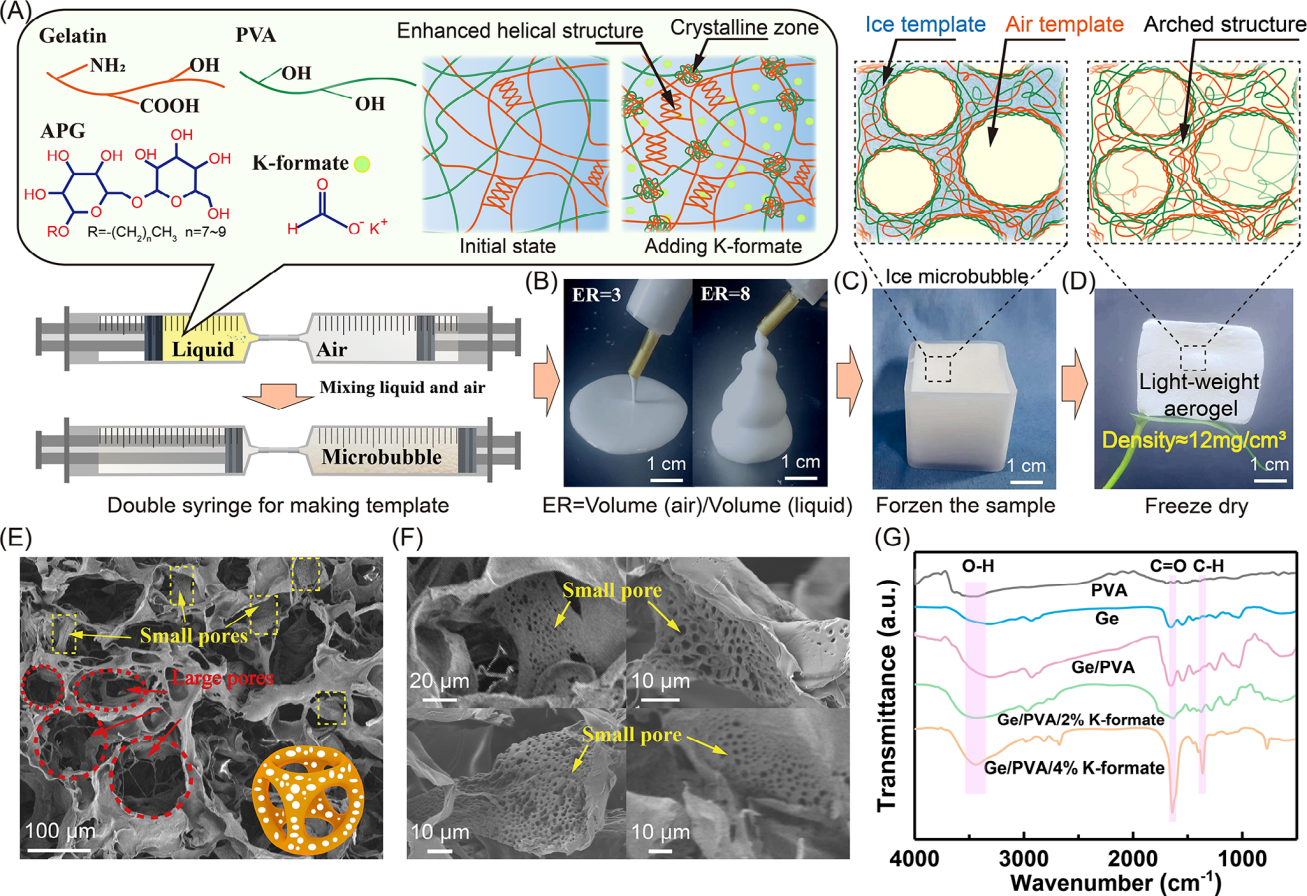
A) Macropore formation mechanism via the dual‐syringe foaming method, where cost‐effective Ge and PVA serve as structural frameworks, whereas flame‐retardant potassium salts induce crystalline domain formation for mechanical regulation. B) Tunable density and rheological properties of bubble templates are achieved by adjusting the gas volume fraction in the syringe. C) Ice template containing bubbles for aerogel formation. D) As‐obtained ultralight aerogel supported by a leaf (ER = 8). E, F) SEM images revealing the macroporous architecture (≈100 µm) and pores of a few µm in size in the aerogel. G) FTIR spectra of various aerogels. Unless otherwise specified, the ratio of Ge to PVA in the aerogel samples was 1:2.

To fabricate superelastic aerogels, we used a dual‐template strategy that enables precise control over aerogel density and mechanical performance. All the raw materials are low‐cost and do not require chemical modification. The preparation protocol involves two critical steps. First, an aqueous Ge solution was blended with PVA at various ratios. Second, 1% alkyl glycoside (APG) was introduced to reduce the dynamic surface tension of the system (Figure S1, Supporting Information), and 2% potassium formate (K‐formate) was added to facilitate crystalline domain formation (**Figure** **1**A). A critical challenge in constructing dome‐shaped architectures lies in the reliable generation of microbubbles. This was addressed by the modified “Tessari” method, which is a foam‐based sclerotherapy technique that was originally developed approximately four decades ago for clinical applications. The modified “Tessari” method employs two syringes connected via flexible tubing (Figure 1A), where the first syringe contains the aqueous phase and the second syringe contains the gas phase.^[^ ^18^ ^]^ Through multiple reciprocating cycles (push‐pull motions) between the syringes, the system achieves the required high shear rates for effective gas‐liquid mixing. Notably, the liquid and air at the syringe outlet undergo spontaneous “exploded” fragmentation into microbubbles during pinch‐off. The foam template (Figure 1B) density can be precisely tuned by adjusting the air fraction while maintaining a consistent bubble size distribution across different air fractions. To the best of our knowledge, this is the only foaming technique in which the air fraction can vary independently of the bubble size distribution. The Tessari method is convenient for us to examine the effect of the ER on the performance of aerogels in the laboratory (with the same average bubble size). The subsequent ice‐templating process involved pre‐freezing the foam at −20 °C (Figure 1C). After lyophilization, aerogels characterized by a hierarchical porous structure are obtained (Figure 1D) at the specific ER range. SEM images (Figure 1E,F) clearly display the voids and interconnect dimensions on the order of a few hundred and tens of µm, respectively. Another interesting feature is the observation of the details of the aerogel walls at higher magnification (Figure 1F). Aerogel walls are characterized by a subporous structure characterized by aligned pores of a few µm in size, as well as rugosity on void walls, which are very suitable for the adhesion of pollutant particles (see Section 2.6). This structure of the aligned pores is driven by the large temperature gradient, which orients the ice crystals during their formation. After freeze‐drying, the oriented ice crystals were removed to produce pores a few µm in size. The resulting architecture demonstrates exceptional mechanical performance, where rigid Ge components serve as reinforcing struts, whereas flexible PVA and crystalline region domains act as elastic nodes, synergistically enabling both high mechanical strength and remarkable elasticity (Movie S1, Supplementary Movie1). Figure 1G shows the FTIR spectra of the four aerogel samples. The broad absorption band observed at 3324 cm^−1^ is attributed to the stretching vibrations of O–H bonds, indicating that the incorporation of K‐formate significantly enhances the crystallinity of PVA and Ge.^[^ ^19^ ^]^ Notably, the characteristic peak at 1348 cm^−1^ exhibited intensified absorption upon the addition of K‐formate, revealing strengthened hydrophobic interactions within the system. Furthermore, the increased intensity of the C═O stretching vibration at 1654 cm^−1^ revealed that the K‐formate facilitated tighter chain entanglement, which contributed to the increased elastic modulus of the template and improved tensile strength of the aerogel (Figures S2 and S3, Supporting Information).

Notably, the Ge/PVA/APG combination was obtained through rigorous screening (Table S1, Supporting Information). We initially developed composite systems incorporating Ge/APG with various polymers (polyurethane, cellulose nanocrystals, cellulose nanofibres, etc.), maintaining fixed concentrations of 5% Ge and 1% APG with an ER of 6 (Table S1, Supporting Information). While all formulations initially exhibited intact structures after lyophilization, most systems suffered structural collapse under compressive/bending stresses. Remarkably, only the Ge/PVA/APG aerogel demonstrated pronounced superelasticity. As shown in Figures S4–S6 (Supporting Information), neither Ge nor PVA only could form stable aerogels because of accelerated bubble coarsening in the foam templates (bubble size increased with time). APG fulfills two critical roles in this system: i) suppressing bubble coarsening kinetics through interfacial stabilization and ii) reducing the dynamic surface tension (Figure S1, Supporting Information) to obtain foams with a high gas‐volume fraction and low density (Figure S7, Supporting Information). The ternary Ge/PVA/APG system maintained foam stability for more than 30 min (Figures S8 and S9, Supporting Information). In stark contrast, the APG‐free Ge/PVA systems exhibited rapid gas‐liquid phase separation within 10 min (Figures S8 and S9, Supporting Information), unequivocally demonstrating the high foam stability required by this dual template strategy.

### 2.2 Morphology, Thermal Insulation Performance and the Cost of Biomass Aerogels with Hierarchical Structures

**Figure 2:**
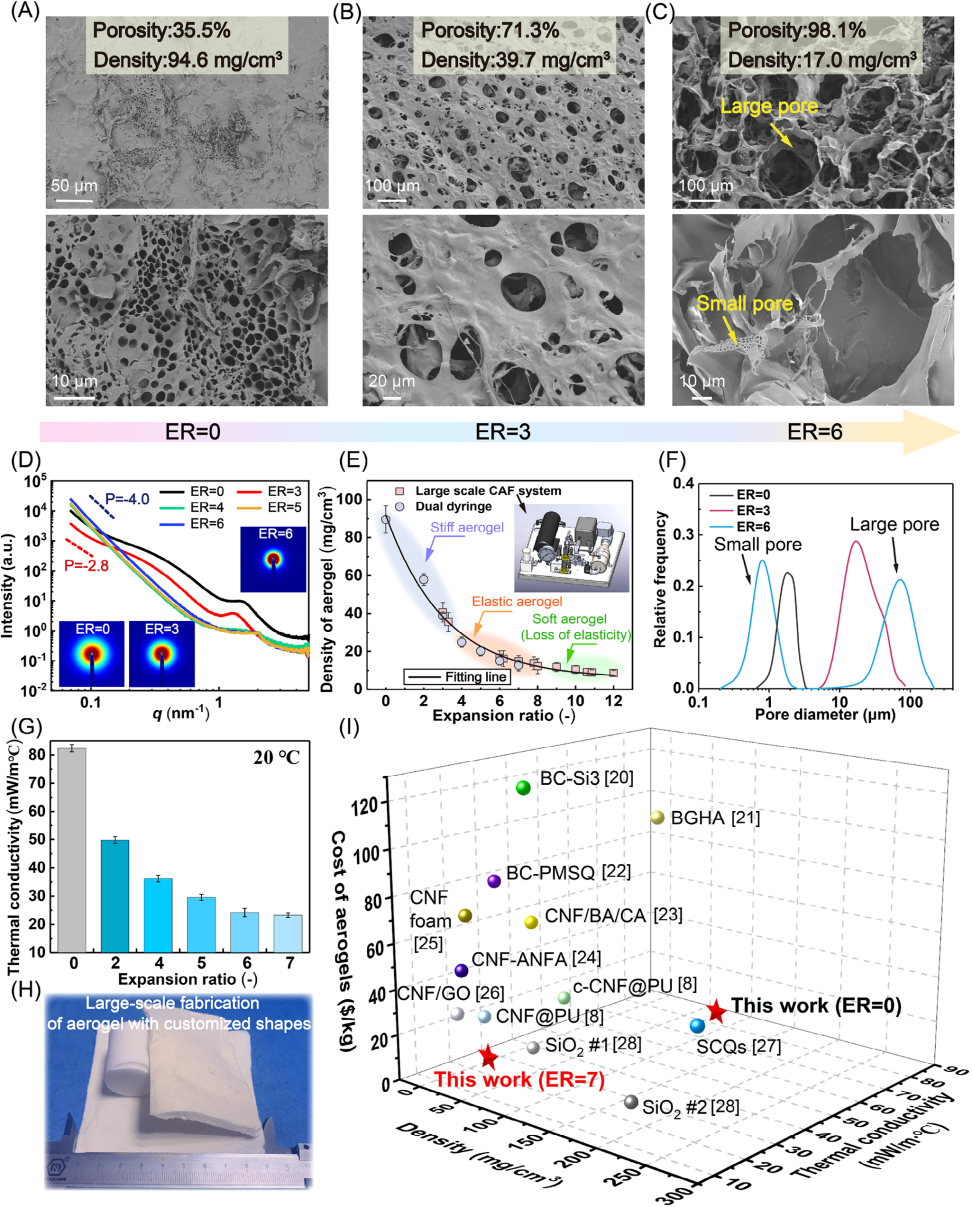
A–C) SEM images of aerogels with different ER values. D) Small‐angle X‐ray scattering (SAXS) profile of the porous aerogel. E) Density comparison of aerogels produced via the dual‐syringe method and customized large‐scale compressed‐air foam system. F) Pore size distributions of aerogels with different ER values. G) Thermal conductivity of aerogels with varying ER. H) Scale‐up preparation process for porous aerogels using the compressed‐air foam system. I) Comprehensive comparison of the thermal conductivity, density, and raw material costs between the aerogels developed in this work and those reported in previous studies.^[^ ^8^, ^9^, ^10^, ^11^, ^12^, ^13^, ^14^, ^15^, ^16^, ^17^, ^18^, ^19^, ^20^, ^21^, ^22^, ^23^, ^24^, ^25^, ^26^, ^27^, ^28^ ^]^ Unless otherwise specified, the ratio of Ge to PVA in the aerogel samples was 1:2.

The dome‐structured network could confer superelasticity to aerogels, as the rigid Ge scaffold anchored by flexible PVA chains enables effective dissipation of external stress. SEM characterization (**Figure** **2**A–C) revealed that both the density and pore architecture of the aerogels are significantly governed by the ER. At ER = 0, only small pores with a peak diameter of 2 µm are observable on the aerogel surface (Figure 2F). A progressive increase in the ER to 6 results in a remarkable porosity enhancement accompanied by a density reduction from 94.6 to 17.0 mg cm^−^^3^, ultimately forming a macroporous network with dome‐shaped structures (porosity >98%). In contrast, the Ge‐free PVA/APG aerogel exhibited neither elasticity nor dome‐like macropores, instead displaying solely fibrous PVA structures (Figure S10, Supporting Information). Qualitative mechanical evaluations revealed that the biobased Ge/PVA/APG aerogel achieved complete shape recovery after the 3000× self‐weight compressive load was removed without structural fracture (Figure S11, Supporting Information).

Structural characterization of aerogel films with different ER values was performed using small‐angle X‐ray scattering (SAXS). The SAXS profiles for different aerogel samples revealed two distinct features: i) a low‐*q* slope variation and ii) a scattering peak position in the 1–2 nm^−1^ range. For ER = 0, the low‐*q* slope (P = −2.8) indicates the presence of entangled PVA‐Ge chains forming a fractal network structure (Figure S12, Supporting Information). Under low ER conditions, the characteristic scattering peak observed at 1–2 nm^−1^ originates from the periodic arrangement of pore spacings. With increasing ER values, the slope transitions to P = −4, accompanied by the movement of the characteristic peak toward the high‐*q* region. These changes are attributed to the formation of dome‐like macropores, resulting in a transition from fractal architecture to discrete spherical structures.^[^ ^29^, ^30^ ^]^

The elasticity and density of aerogels are significantly influenced by the ER (Figure 2E; Figure S13, Supporting Information). The experimental data revealed a rapid decrease in aerogel density with increasing ER (Figure S13, Supporting Information), reaching a minimum value of 9 mg cm^−^^3^ at ER>10. Our findings revealed that a large ER results in a complete loss of elasticity (Figure 2E; Figure S14, Supporting Information), whereas a small ER results in excessive rigidity (Figure 2E; Figure S14, Supporting Information). The optimal elasticity was observed within the ER range of 4–8 (Figure S14, Supporting Information), which is slightly greater than the critical ER≈2.78 required for the bubble jamming transition in 3D foam systems. Notably, the elastic behavior of the aerogel originates from the jammed state of the bubbles in the foam template. At ER = 2.2 (Figure S15, Supporting Information), the bubbles in the template are spherical and do not touch. At ER = 12.7 (Figure S15, Supporting Information), bubbles exhibit a polyhedral morphology, whereas the bubbles touch and take the shape of a squashed sphere at each bubble/bubble contact at ER = 7.4 (Figure S15, Supporting Information), enabling the formation of well‐defined dome‐like structures in freeze‐dried aerogels. The scalable production of superelastic aerogels is facilitated by a compressed air foaming system (Figure S16, Supporting Information), as shown in Figure 2E,H. Compared with the Tessari method, compressed air foaming produces bubbles with greater polydispersity and a larger average size (Figure S17, Supporting Information). Remarkably, the thermal conductivity dramatically decreases with increasing ER, achieving an ultralow value of 24 mW/(m °C) at ER = 6. The optimal thermal conductivity was achieved when the Ge:PVA ratio reached 1:1 or 1:2 (Figure S18, Supporting Information). A lower thermal conductivity was also maintained in the system even under low‐temperature conditions (‐20 °C, Figure S19, Supporting Information). This exceptional thermal insulation stems from the multiscale porous architecture created by dual templating (air and ice templates), which establishes intricate tortuous pathways and numerous solid‒gas interfaces. Furthermore, the densely interconnected nodes (the ER exceeds the ER at the bubble jamming transition point) within the dome‐like network generate additional solid‐gas and solid‐solid interfaces. These interfaces effectively increase phonon scattering, thereby significantly suppressing heat transfer through the aerogel matrix.

To highlight the comprehensive advantages of the as‐prepared elastic bio‐based aerogel, we conducted a comparative analysis of its density, thermal conductivity, and raw material costs with those of previously reported aerogels (Figure 2I). First, the Ge/PVA/APG aerogel exhibits exceptionally low thermal conductivity (24 mW/(m °C)), whereas most cellulose‐based aerogels exhibit thermal conductivity values of ≈30 mW/(m °C) or higher. Furthermore, the Ge/PVA/APG aerogel possesses remarkably lighter structural characteristics than other cellulose aerogels do, with tunable density and elasticity. Notably, the developed Ge/PVA/APG aerogel has substantial cost advantages, with raw material expenses of approximately USD 5.24/kg, in stark contrast to conventional CNF‐based aerogels, which typically cost USD 30–100/kg (Table S3, Supporting Information). In conclusion, the elastic Ge/PVA/APG aerogel has superior integrated performance advantages and cost‐effectiveness compared with existing cellulose‐based aerogels.

### 2.3 Hyperelasticity of Biomass Aerogel with Hierarchical Structure

**Figure 3:**
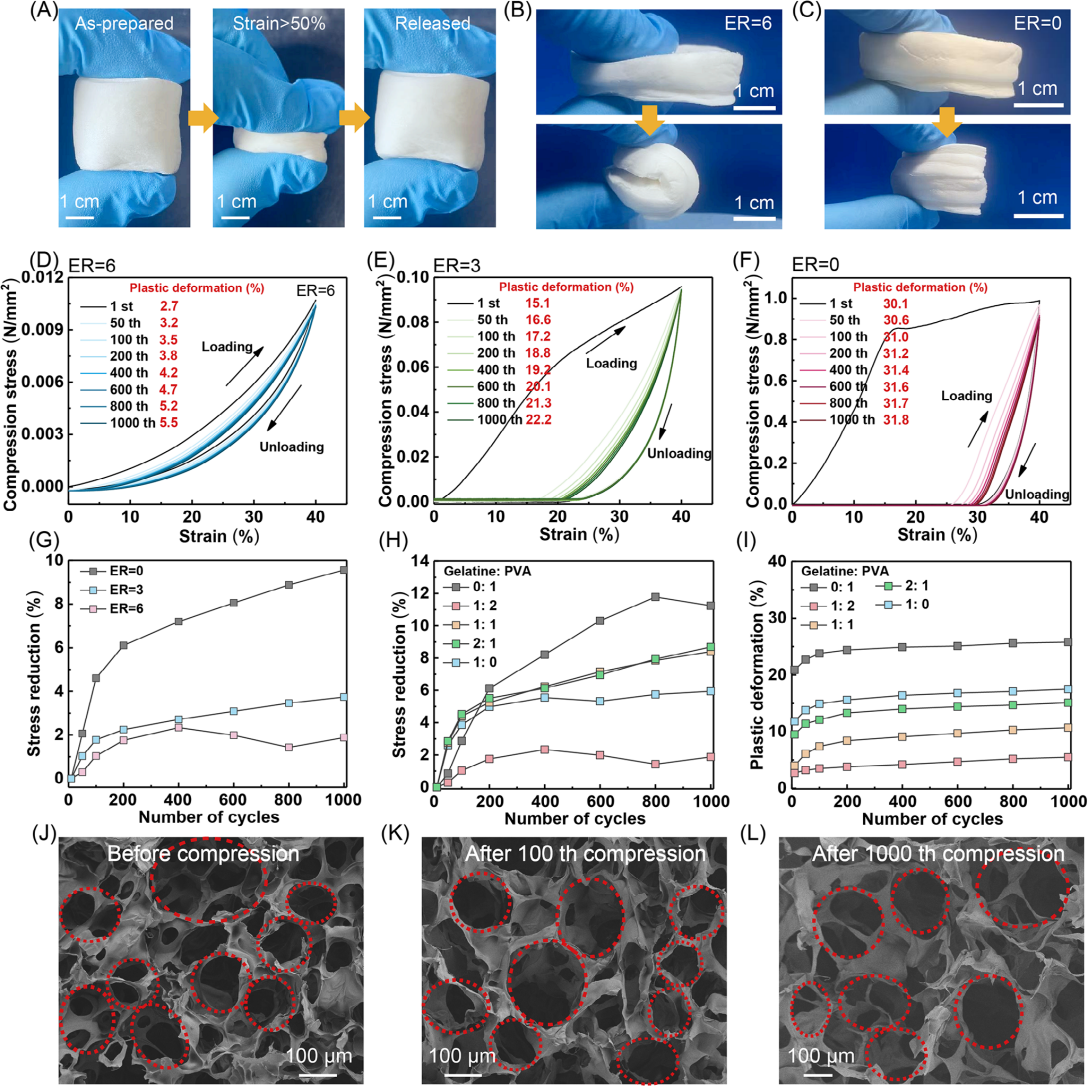
A) Compressive resilience of the Ge/PVA/APG (Ge:PVA = 1:2) aerogels at ER = 6. B, C) Comparative photographs of Ge/PVA/APG aerogels with ER = 6 and ER = 0 before and after folding. D–F) Compression stress‐strain curves (1000 cycles at 40% strain) of the aerogel samples under varying ER conditions. G) Stress reduction during cyclic compression testing of Ge/PVA/APG aerogels across different ER values. H, I) Effects of the Ge‐PVA ratio on stress reduction and plastic deformation during 1000 cycles of compression testing (ER = 6). J–L) SEM images of pore architecture evolution in the Ge/PVA/APG aerogel after 100 and 1000 compression cycles. Unless otherwise specified, the ratio of Ge to PVA in the aerogel samples was 1:2.

Hierarchical porous architecture endows Ge/PVA/APG aerogels with exceptional compressive‐bending recovery capabilities through effective local strain mitigation (**Figure** **3**A,B). For the aerogel with ER = 0, structural failure manifests as irreversible shape deformation and crack propagation upon bending (Figure 3C). Remarkably, the ER = 6 aerogel achieves merely a 1.9% stress reduction and 5.5% residual strain after 1000 compression cycles at 40% strain (Figure 3D), confirming its outstanding elastic recovery and fatigue resistance. This superior compressible elasticity originates from the aerogel's unique dome‐shaped macropore structures and high porosity (98.1%, Figure 2C). Mechanistic studies reveal that the mechanically robust spherical dome framework effectively absorbs and dissipates external stress energy through large‐scale self‐deformation. The microstructural analysis confirmed the well‐preserved dome morphology and hierarchical structure even after 1000 compression cycles (Figure 3J–L), corroborating its structural elasticity. In contrast, control samples with ER = 0 and ER = 3 achieve compromised elastic performance, as evidenced by significant stress reduction (6% at the 200th cycle, 8% at the 600th cycle for ER = 0, Figure 3G) and permanent pore structure collapse with notable plastic deformation in ER = 3 samples after 100 cycles (Figure S20, Supporting Information).

To systematically investigate the correlation between the composition and compressive elasticity of aerogels, we conducted a comprehensive study on the effect of the Ge‐to‐PVA ratio with a fixed ER (ER = 6), as shown in Figure S21 (Supporting Information) and Figure 3H,I. The results indicated that neither the Ge/APG (Ge: PVA = 1:0) system nor the PVA/APG (Ge: PVA = 0:1) system can produce superelastic aerogels. Specifically, the Ge/APG aerogel (Ge: PVA = 1:0) exhibited rigid characteristics with irreversible deformation and high plastic strain upon loading, whereas the Ge/PVA aerogel (Ge: PVA = 0:1) showed excessive softness, retaining 25.8% residual strain after 1000 compression cycles (Figure 3I). Through composition optimization, the optimal Ge/PVA ratios of 1:1 and 1:2 were identified to achieve superior superelasticity, as evidenced by significantly smaller hysteresis loops in their loading‐unloading curves (Figure S21, Supporting Information) than those of the other formulations, indicating increased energy dissipation capability through hierarchical structures. Microstructural analysis revealed the underlying mechanism: excessive PVA content induced skeleton adhesion and structural collapse (Figure S22B, Supporting Information), whereas pure Ge/APG systems formed rigid walls susceptible to permanent fracture during compression (Figure S22A, Supporting Information). In contrast, the dome‐shaped architecture in the Ge/PVA composites demonstrated remarkable elasticity, effectively dissipating the stress concentration through localized deformation to prevent structural failure. The synergistic combination of hierarchical porosity and a dome‐like microstructural configuration plays a critical role in achieving the exceptional superelasticity of Ge/PVA aerogels.

### 2.4 Repairability, Recyclability, and Biodegradability of Biomass Aerogels with Hierarchical Structures

**Figure 4:**
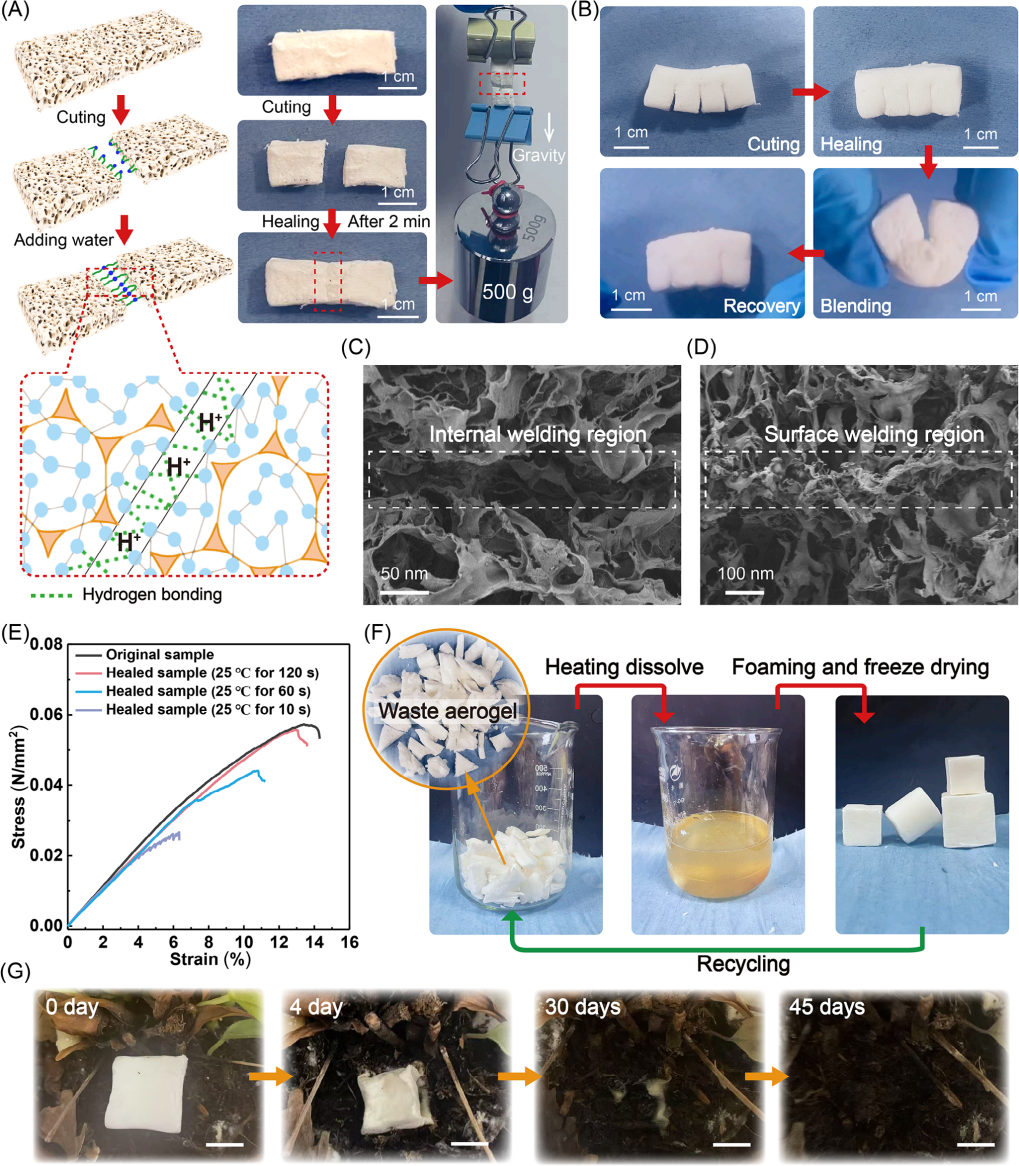
A) Self‐healing capability of the superelastic Ge/PVA/APG aerogel through dynamic hydrogen bonding interactions. The healed aerogel sustains tensile loads exceeding 6000 times its own weight. B) The multisection fractured Ge/PVA/APG aerogel exhibited resilient elastic recovery upon bending deformation after self‐healing. C, D) SEM images of internal and external welding interfaces in self‐healed Ge/PVA/APG aerogels. E) Tensile stress‐strain curves of the Ge/PVA/APG aerogels after various healing durations. F) Schematic of the closed‐loop recycling protocol for Ge/PVA/APG aerogel samples. G) Biodegradation process of Ge/PVA/APG aerogel samples in soil environments (the scale bar represents 12.5 mm). Unless otherwise specified, the ratio of Ge to PVA in the aerogel samples was 1:2.

In pursuit of extended service life for aerogels, the integration of self‐healing capabilities holds paramount significance. This endeavor remains a formidable challenge given the inherent porous architecture of aerogels. Our Ge/PVA/APG aerogel distinguishes itself through the incorporation of dynamic hydrogen bonds (Figure S23, Supporting Information) within the cross‐linked network, which confers exceptional water‐activated self‐healing properties. The healing protocol involves bisecting the aerogel with a blade. The subsequent application of deionized water to one fractured interface, followed by precise alignment and contact with the complementary dry surface, enables complete structural restoration within 2 min under ambient conditions. Notably, the healed aerogel (0.08 g) exhibited sufficient mechanical strength to withstand a tensile load of 500 g without fracture, which was more than 6000 times its own mass (**Figure** **4**A). Multiple segment‐cutting experiments further validated this self‐repair mechanism, where water‐assisted welding restored structural integrity to permit elastic recovery after bending deformation (Figure 4B). Morphological analysis of the healed interfaces revealed great integration between the internal weld region and the external weld region, confirming microstructural healing (Figure 4C,D). Quantitative assessment through tensile testing demonstrated rapid healing kinetics, with the repaired samples recovering more than 90% of the original tensile strength within merely 2 min. This accelerated macroscopic healing mechanism stems from the abundance of hydrophilic groups at the fracture interfaces of the aerogels. When the dry surface and wetted surface contact, the exposed Ge and PVA chains on the aerogel surface link through hydrogen bonding interactions, leading to the reconstruction of the aerogel network. FTIR spectra prove this hydrogen bonding interaction (Figure S23, Supporting Information). Upon hydration, exposed Ge/PVA/APG molecular chains undergo rapid dissolution and re‐entanglement, driving efficient structural reintegration.

The recyclability of aerogels represents a remarkable characteristic that significantly extends their service life. Given the abundant hydrogen‐bond interactions within the Ge/PVA/APG aerogel network, this study employs hot water treatment to depolymerize the dynamic cross‐linked networks, thereby establishing an eco‐friendly recycling protocol. The experimental results reveal that end‐of‐life Ge/PVA/APG aerogels can be completely converted to homogeneous foaming solutions through hierarchical network depolymerization when they are heated to 60 °C or above in hot water. The recovered solution undergoes cooling, secondary foaming, and freeze‒drying processes to achieve precise structural reconstruction, forming a closed‐loop recycling system. To validate the reprocessability, we successfully reconstituted waste aerogel fragments into various geometries, including cuboids and cylinders. Remarkably, the recycled aerogels (Figure S24, Supporting Information) maintain comparable tensile strength (0.059 MPa), thermal conductivity (23.9 mW/(m °C)), and elastic recovery to their pristine counterparts. In addition, after repeated recovery‐regeneration cycles and long‐term thermal exposure (Figure S25, Supporting Information), the performance of the regenerated aerogels differed slightly from that of the original sample, confirming the retention of its performance. This nondestructive reprocessing strategy enables the circular utilization of elastic aerogels with near‐perfect recovery efficiency (≈100%) and well‐preserved performance, rendering Ge/PVA/APG aerogels particularly attractive for sustainable applications.

Compared with conventional lightweight materials such as bioplastics or polyurethane foams, which require multiple enzymes and months to years for gradual decomposition, Ge/PVA/APG aerogels exhibit targeted and accelerated degradation characteristics. To demonstrate this property, we conducted a soil exposure experiment in Hefei with a 2.5×2.5×2.5 cm^3^ aerogel sample. As shown in Figure 4G, microbial activity from bacteria and fungi progressively attacked and digested the aerogel structure, resulting in complete fragmentation and ultimately biodegradation into carbon dioxide and water within 45 days. Comparative analysis of aerogel's FTIR spectra before and after degradation reveals substantial attenuation in peak intensities corresponding to C═O (amide I band in Ge), C‐N (amide II bands in Ge), C‐O (bands in PVA and Ge), and C‐O‐C bonds (ether linkage in glycoside of APG) within the degraded specimens relative to their pristine counterparts, as shown in Figure S26 (Supporting Information). The above evidence conclusively confirms the aerogel's inherent biodegradability. Notably, this aerogel maintains exceptional structural stability in non‐degradative environments, retaining its original density (Figure S27, Supporting Information) for over a year under typical ambient humidity conditions (Hefei, annual average RH: 68%). Below 80% relative humidity (RH), humidity had negligible effects on the compressive strength and thermal conductivity of Ge/PVA/APG aerogels (Figure S28, Supporting Information). Remarkably, applying a commercial hydrophobic polydimethylsiloxane (PDMS) coating can significantly enhance moisture resistance. Even at 90% RH, the modified aerogels maintained robust elasticity (Figure S28C, Supporting Information), low thermal conductivity (Figure S28D, Supporting Information), and structural integrity of pores (Figure S28F, Supporting Information). Long‐term testing under 90% RH confirmed that the PDMS‐coated aerogels retained low thermal conductivity after 96 h of exposure (Figure S28E, Supporting Information), demonstrating exceptional humidity stability.

In a word, the environmental credentials of the aerogel are improved by its closed‐loop recyclability, enabling theoretically infinite reuse cycles through damage‐free recovery processes. Moreover, the controllable degradation profile allows the aerogel to reintegrate safely with nature as benign waste, thereby completing a fully sustainable lifecycle from production to final disposal.

### 2.5 Excellent Flame Retardancy of Biomass Aerogels with Hierarchical Structures

**Figure 5:**
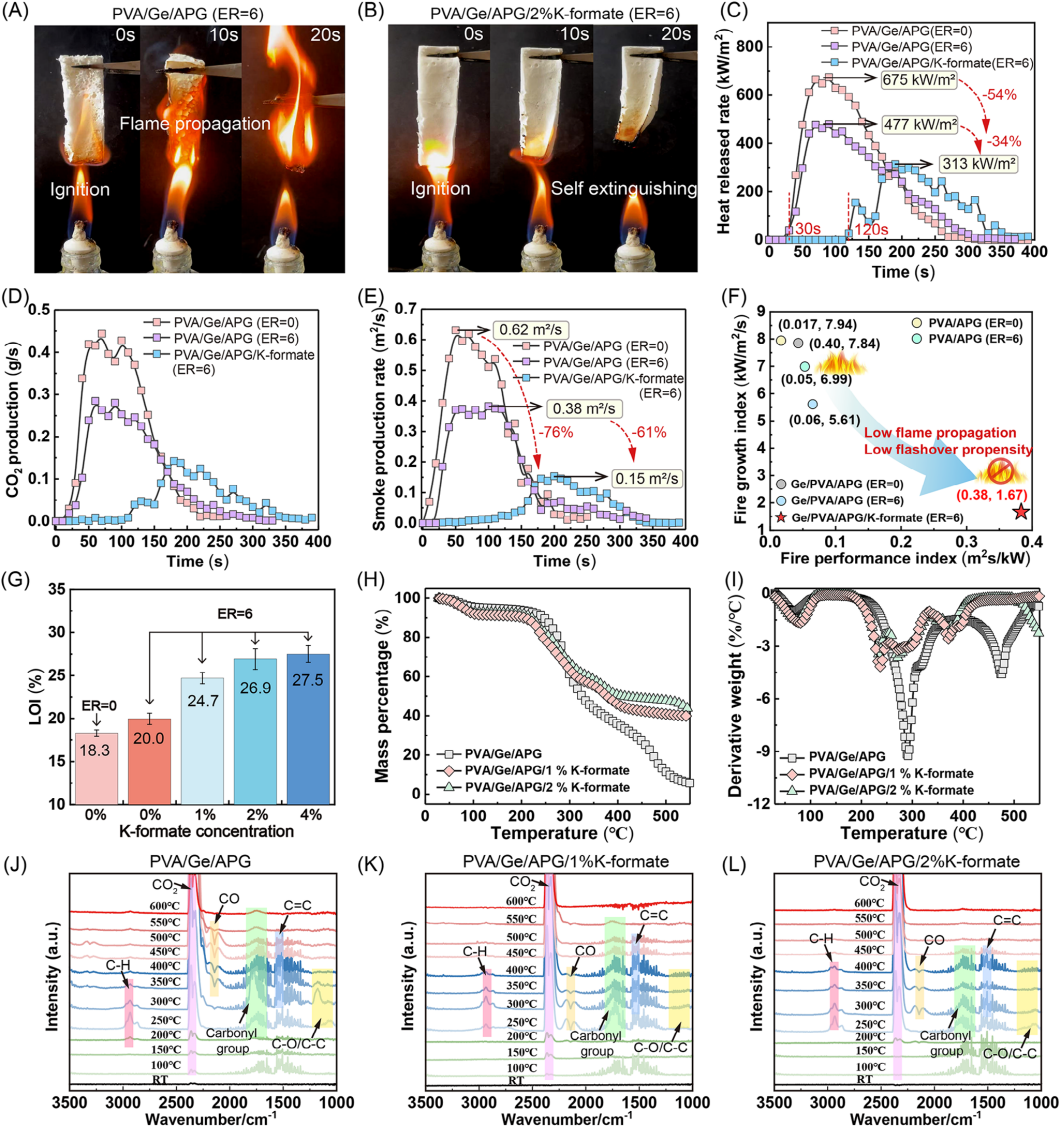
A, B) Flammability test of the Ge/PVA/APG aerogel (ER = 6) with and without the K‐formate. C‐E) Heat release rates, CO_2_ production rates, and smoke production rates of the aerogel samples during combustion exposed to 35 kW m^−^^2^ radiant heat flux. F) FGI and FPI of the aerogel samples. G) LOI of the aerogel samples. H, I) TG and DTG curves of the aerogel samples. J–L) FTIR spectra of the pyrolysis products of the aerogel samples. Unless otherwise specified, the ratio of Ge to PVA in the aerogel samples was 1:2.

Surprisingly, the bio‐based aerogel reported in this study has self‐extinguishing properties, in contrast with the high flammability typically observed in most previously reported organic aerogels.^[^ ^14^, ^15^, ^16^ ^]^ When it is ignited by an alcohol lamp flame, the bio‐based aerogel exhibits spontaneous flame extinction (Figure 5B). This remarkable self‐extinguishing behavior is attributed to the synergistic effects of potassium salt incorporation and the optimized ER. Notably, the control samples without the addition of potassium salt under identical ignition conditions exhibited complete combustion with flame propagation throughout the sample (**Figure** **5**A). Cone calorimetry tests were conducted to systematically evaluate the combustion characteristics under controlled radiant heat flux (35 kW m^−^^2^). Critical fire parameters, including the time to ignition (*t*_ign_), heat release rate (HRR), smoke production rate (SPR), and CO₂ yield (CO₂P) were quantitatively analyzed. The Ge/PVA/APG rigid aerogel (ER = 0) exhibited a peak HRR (pHRR) of 675 kW m^−^^2^. Remarkably, the development of hyperelastic aerogels with increased ER = 6 demonstrated a significant 54% reduction in the pHRR (477 kW m^−^^2^). Further enhancement through potassium salt modification resulted in an additional 34% decrease in the pHRR (Figure 5C). The temporal profiles of CO₂ emissions and smoke production paralleled the HRR trends (Figure 5D,E). Optimization of ER (0→6) combined with potassium salt modification resulted in 76% suppression of smoke generation.

Two critical fire safety indices were employed for quantitative risk assessment:^[^ ^31^, ^32^, ^33^ ^]^ the fire performance index and the fire growth index (Equation (1) and (2)). Compared with the Ge/PVA/APG sample (ER = 0), the superelastic Ge/PVA/APG/K‐formate aerogel (ER = 6) achieved superior FPI (0.38 m^2^s/kW) and FGI (1.67 kW/m^2^/s) values, representing 9.5‐fold enhancement and 4.6‐fold reduction, respectively, over those of the Ge/PVA/APG sample (Figure 5F). Significantly, potassium ion incorporation increased the limiting oxygen index (LOI) of the aerogel to 27.5% (Figure 5G), exceeding the critical threshold for self‐sustained combustion under normal atmospheric conditions. These comprehensive results substantiate that the integration of potassium salts with structure optimization effectively establishes an intrinsic flame‐retardant mechanism in our bio‐based aerogels.

(1) $$FPI=\frac{t_{ign}}{PHRR}$$

(2) $$FGI=\frac{PHRR}{t_{PHRR}}$$

TG‐FTIR analysis was employed to elucidate the flame‐retardant mechanism of the superelastic aerogel. For the aerogel sample without K‐formate, the first weight loss peak occurred at 30–100 °C (Figure 5I), primarily attributed to the evaporation of water bound to hydrophilic groups on Ge, PVA, and APG. The main pyrolysis temperature range of the aerogel was observed between 200–350 °C (Figure 5I), resulting from Ge decomposition into polypeptides, cleavage of glycosidic bonds (leading to L‐glucose formation and combustible volatile release), and PVA degradation. Figure 5J,K shows the FTIR spectra of gaseous pyrolysis products at different temperatures. Peaks in the 1250–1050 cm^−1^ range were assigned to C‐O/C‐C stretching vibrations. The characteristic peak at 2300–2400 cm^−1^ corresponds to CO_2_ vibration, generated through cleavage and reorganization of carboxyl (‐COOH) and carbonyl (C═O) groups. Multiple bands detected in the 1800–1700 cm^−1^ region indicated the release of carbonyl‐containing products: The 1795 cm^−1^ peak derived from carbonyl/ester group‐containing products. Peaks at 1761 and 1746 cm^−1^ originated from saturated and unsaturated ketones, respectively. The 1725 cm^−1^ peak corresponded to saturated aldehydes. The 1710 cm^−1^ peak arose from unsaturated aldehydes. Between 250–350 °C, the high‐intensity C═C peaks and low‐intensity C‐H stretching at 2930 cm^−1^ suggested aromatic species release. Volatiles from the aerogels were essentially fully released below 350 °C. Above 400 °C, the 2250–2150 cm^−1^ peak indicated CO generation, primarily from solid carbonaceous residue cleavage. Secondary cracking of esters, carbonyl groups, and volatiles also contributed to CO release.

The thermogravimetric curves (Figure 5H) revealed a significant increase in residue yield upon K‐formate incorporation. While the control sample (without K‐formate) exhibited minimal residual carbon (<4%) at 600 °C, the aerogel containing 1% K‐formate demonstrated a remarkable char residue of ≈40%, highlighting the exceptional catalytic carbonization effect of K‐formate. The addition of K‐formate induced a red shift in the main C═O peak wavenumber of aerogel pyrolysis products (Figure 5K,L), indicating a compositional shift from carboxylic acids to predominantly aldehydes/ketones in carbonyl‐containing species. Concurrently, significant attenuation of C‐O/C‐C peak intensities (Figure 5K,L) suggested reduced generation of ether compounds in pyrolysis products. The abundant polar alcoholic hydroxyl and amino groups on the glucose units of APG underwent catalytic dehydration reactions mediated by K‐formate. The resulting ketonic/aldehyde intermediates subsequently undergo further dehydration under pyrolysis conditions to form conjugated enone structures,^[^ ^34^, ^35^, ^36^ ^]^ which preferentially participate in crosslinking reactions to generate thermally stable char in low‐to‐medium temperature ranges. Notably, the initial release temperature of CO decreased substantially, likely attributed to the early cracking reaction catalyzed by K‐formate. The above results suggested that nucleophilic potassium salts facilitate the cleavage and reorganization of protein carboxyl and carbonyl groups, effectively reducing the pyrolysis temperature while increasing char formation. Derivative thermogravimetric (DTG) analysis (Figure 5I) demonstrated a substantial decrease in the maximum pyrolysis temperature from 295 °C (control) to 230 °C with K‐formate incorporation. Broido equation^[^ ^37^ ^]^ calculations revealed a 29% reduction in the activation energy (Ea) for the primary thermal degradation stage—from 62.45 kJ mol^−1^ for Ge/PVA/APG to 35.17 kJ mol^−1^ for the K‐formate modified system (Figure S29, Supporting Information). The ratio of peak D to peak G is usually used to evaluate the degree of graphitization of residual carbon.^[^ ^38^, ^39^, ^40^ ^]^ The decrease in the intensity ratio of the D and G peaks via Raman spectroscopy indicates that the addition of K‐formate results in an increase in the degree of graphitization of residual carbon (Figure S30, Supporting Information). These structural insights demonstrate that K‐formate modification lowers the principal thermal decomposition threshold of biomass aerogels. The modified pyrolysis pathway promotes intermediate conversion to flame‐retardant char while establishing an effective thermal‒oxygen barrier, ultimately enabling self‐extinguishing behavior.

### 2.6 Radiation Cooling and Smoke Filtration Applications of Hyperelastic Biomass Aerogels

In addition to exceptional flame retardancy, Figure S31 (Supporting Information) demonstrates that as the ER increases from 0 to 6, the aerogel achieves a solar reflectance exceeding 97%. For the aerogel with ER = 6, the average cooling effect was ≈3.2 °C during the cloudy period, whereas during the sunny period, the cooling effect increased to 12.5 °C (Figure S32, Supporting Information), highlighting its significant potential as an energy‐efficient building material for radiative cooling applications (Figure S33, Supporting Information). To enhance water resistance, Ge/PVA/APG superelastic aerogels can be treated with commercial hydrophobic coatings such as PDMS. The modified aerogel maintains excellent structural integrity after complete water immersion (Figure S34, Supporting Information) while retaining nearly unaffected elasticity (Movie S2, Supplementary Movie2) and solar reflectivity (Figure S31, Supporting Information). We also highlighted the potential application of superelastic aerogels in smoke capture. The device used for the filtration test of the aerogel samples is shown in Figure S35. The porous structure enables the Ge/PVA/APG aerogel to function as a biodegradable air filtration material that withstands high airflow rates without structural collapse. Figures S35–S37 (Supporting Information) showed the smoke particle capture performance of aerogels (thickness = 0.5 cm) compared with that of commercial activated carbon masks. Under a maximum airflow of 5 L min^−1^, the superelastic aerogel in this work exhibited exceptional smoke removal efficiency (Figure S36, Supporting Information) and a low filtration pressure drop (<70 Pa, Figure S37, Supporting Information), significantly outperforming commercial masks. This finding indicates outstanding smoke particle capture capability and structural robustness. This aerogel represents a crucial advancement beyond conventional high‐cost, cellulose‐based counterparts, offering a unique combination of cost‐effectiveness, high solar reflectance, superior flame retardancy, and exceptional char‐forming capability. These synergistic properties make it a groundbreaking material for sustainable energy conservation applications and advanced fire safety scenarios.

The above results show that the Ge/PVA/APG aerogel has significant potential for applications in energy‐efficient construction and fire safety materials. However, its practical implementation requires overcoming challenges related to environmental resistance and mechanical durability. For example, while the material exhibits exceptional solar reflectance reaching 97% (ER = 6), real‐world performance may be compromised by climatic conditions such as atmospheric humidity and particulate deposition. Furthermore, sustained outdoor deployment necessitates the consideration of surface self‐cleaning properties. Although the aerogel has effective smoke particle entrapment capabilities, the regeneration of the aerogel after smoke capture remains a critical challenge requiring resolution.

## 3 Conclusion

This study used a dual‐template strategy (ice‐ and bubble‐templating) for fabricating ultralight, superelastic biomass aerogels with hierarchical architectures. By synergistically integrating Ge, PVA, APG, and K‐formate, the aerogels achieve exceptional mechanical elasticity (5.5% residual strain after 1000 compressive cycles at 40% strain), ultralow density (12 mg cm^−3^), and outstanding thermal insulation (24 mW/(m °C)). The macropores (≈100 µm) and small pores of a few µm in size, engineered via microbubble jamming and ice‐templating, enable efficient stress dissipation through localized deformation. We found that the elastic behavior of the aerogel originates from the jammed state of the bubbles in the foam template. A large ER results in a complete loss of elasticity, whereas a small ER results in excessive rigidity. The optimal elasticity of the aerogel was observed within the ER range of 4–8, which is slightly greater than the critical ER≈2.78 required for the bubble jamming transition in the 3D foam template. The aerogel demonstrated remarkable self‐healing ability (90% strength recovery in 2 min) and closed‐loop recyclability (≈100% efficiency) via water‐assisted reprocessing due to the presence of the dynamic hydrogen‐bonded network. Furthermore, potassium salt modification endows intrinsic flame retardancy (LOI = 27.5%, 76% reduction in the smoke production rate) by catalyzing char formation and establishing thermal‒oxygen barriers. We also highlighted the potential application of superelastic aerogels in smoke capture and radiative cooling. Combined with its cost‐effectiveness (USD 5.24/kg), scalable production, and full biodegradability, this aerogel outperforms conventional cellulose‐based aerogels (USD 30–100/kg) in terms of integrated performance, offering transformative potential for sustainable thermal insulation, fire‐safe materials, and energy conservation technologies.

Although the use of low‐cost Ge and PVA in this study can significantly reduce the raw material costs of aerogels, the energy consumption during processing (particularly the freeze‐drying step) remains a significant factor constraining production costs. Future research will focus on improving aerogel preparation techniques, such as developing ambient‐pressure‐dried preparation technologies for superelastic aerogels. In this technical pathway, the critical point lies in the optimal selection of suitable cross‐linking agent systems to avoid local stress concentrations, ensuring good crack resistance.

## 4 Experimental Section

### Material

Gelatine (240 Bloom, abbreviated as Ge) and polyvinyl alcohol (abbreviated as PVA, type 2448) were purchased from Macklin Biochemical Technology Co., Ltd, China. K‐formate was also obtained from the same supplier. Commercial polydimethylsiloxane (PDMS) coatings were purchased from Kraft Company. Alkyl polyglycoside (abbreviated as APG, 50% purity with critical micelle concentration of 0.5%) was procured from Shanghai Fine Chemical Co., Ltd.

### Preparation of the Biomass Aerogel

Through multiple experimental explorations, the compositional formula of the aerogel was determined, with the composition of the aerogel shown in Table S1 (Supporting Information). First, 10 wt.% Ge solution and 4 wt.% PVA solution were prepared separately. Subsequently, the Ge solution was mixed with the PVA solution at a mass ratio of 1:2 to form a Ge/PVA solution. Specific amounts of APG (1 wt.%) and K‐formate (2 wt.%) were then added to the Ge/PVA solution under stirring to create the Ge/PVA/APG/K‐formate solution. To investigate the effects of PVA‐to‐Ge ratios on aerogel performance, solutions with different PVA/Ge proportions were also prepared. An improved Tessari method was employed to fabricate bubble templates. This modified technique utilizes two syringes connected by flexible tubing 30 mL medical syringe (HongDa Company) containing the Ge/PVA/APG/K‐formate solution and another containing air. High shear rates required for effective gas‐liquid mixing were achieved through multiple reciprocating cycles (push‐pull motions) between the syringes. The solution‐air mixture at the syringe outlet spontaneously underwent “explosive” fragmentation into microbubbles. By adjusting the ER (= gas volume/liquid volume), the template density could be precisely regulated. The Tessari method is convenient for us to study the effect of ER on the performance of aerogels under constant bubble size in the laboratory. We transferred the foam template obtained into a silicone mold using the syringe, then froze it in a −20 °C refrigerator for 5 h to form an ice template. The frozen sample was freeze‐dried for 48 h using an FD‐1A‐50 freeze dryer (BIOCOOL Co., Ltd., China) to obtain the final hyperelastic aerogel sample.

### Characterizations

The pore structure of the aerogel was examined using field emission scanning electron microscopy (FE‐SEM, ZEISS Sigma 360, and Gemini SEM500). The bubble dimensions of the foam template were characterized with an optical microscope (MV3000, JiangNan). The dynamic surface tension of the liquid at different bubble lifetimes was measured using a dynamic surface tensiometer (BPA‐2P, SINTERFACE). The foaming capacity of various liquids was evaluated via the Ross‐Miles method. Oscillatory rheological properties of liquids and foams were determined using a TA rheometer (Discovery HR‐2, 25 mm parallel plate geometry with 2 mm gap). Structural information of aerogel samples was obtained using an Anton Paar SAXSpoint_2.0 small‐angle X‐ray scattering system. Compression (loading rate: 2 mm min^−1^) and tensile (loading rate: 1 mm min^−1^, 1000‐cycle compression test) properties of aerogels were assessed using an AGS‐X‐50N universal testing machine (Shimadzu, Japan). Limiting oxygen index (LOI) measurements were conducted on an FS8500 oxygen index tester (SIFANG Instrument Co., China). Combustion performance was evaluated using an ICONE cone calorimeter (Fire Testing Technology), where control and treated samples (100 × 100 mm^2^) were exposed to 35 kW/m^2^ heat flux. The thermal conductivity of aerogels at different temperatures was measured with a Hot Disk TPS 2500S thermal conductivity analyzer. Thermal stability and gaseous decomposition products were investigated by thermogravimetric‐Fourier transform infrared spectroscopy (TG‐FTIR, PerkinElmer TGA4000, and SP2) at a heating rate of 10 °C min^−1^. FTIR spectra of aerogels were acquired using a Thermo Fisher Scientific Nicolet iS20 spectrometer across the wavenumber range of 500–4000 cm^−1^. UV/visible/near‐infrared diffuse reflection tests of the aerogel samples were performed on a Shimadzu UV‐3600I Plus instrument (Shimadzu, Japan).

## Conflict of Interest

The authors declare no conflict of interest.

## Supporting information



Supporting Information

Supplementary Movie1

Supplementary Movie2

## Data Availability

The data that support the findings of this study are available from the corresponding author upon reasonable request.
