# Supplementary material for: Superelastic Fire‐Safe Aerogel with Hierarchical Structures via Dual Templates Aided by Microbubble Engineering
Source: Adv Sci (Weinh). 2025 Jun 19;12(34):e06808. doi: 10.1002/advs.202506808 (PMC12442648; doi:10.1002/advs.202506808)
Supplement: Supplementary file 1 — Supporting Information [file ADVS-12-e06808-s002.docx]

Supplementary Material
Superelastic Fire-Safe Aerogel with Hierarchical Structures via Dual Templates Aided by Microbubble Engineering
Xiaoyang Yua, Huan Lia, Ning Kanga, Shouxiang Lua, Mingjun Xua,b,*, Man Pun Wanb
State Key Laboratory of Fire Science, University of Science and Technology of China, Hefei 230026, China
School of Mechanical and Aerospace Engineering, Nanyang Technological University, Singapore 639798, Singapore
E-mail: mingjun.xu@ntu.edu.sg (Mingjun Xu)


S1 Adsorption kinetics calculation using the Rosen's approach
In this study, the Rosen's approach is used to fit the dynamic surface tension curves [1]. The Rosen's equation is described as follows:
		(S1)

Here, $\gamma$(t) is the dynamic surface tension; $\gamma_{0}$ is the equilibrium surface tension of water (~75 mN/m); $\gamma_{m}$ is the meso-equilibrium surface tension at t=10000 ms; t is the bubble lifetime. t* is a constant with the dimensions of time. The t* value depends on the diffusion rate of molecules from the bulk solution to the subsurface. The lower the t* value, the lower the hindrance of diffusion, or the faster the diffusion rate of added molecules. The n is a constant, depending on the molecular structure. It indicates the difference between the energies of adsorption and desorption of molecule. The higher the n value, the higher the potential barrier of adsorption or the greater the difficulty for molecules to adsorb at the air-liquid interface. Eq. (S1) can be simplified as follows:
		(S2)

Eq. (S3) can be obtained after taking the logarithm on both sides of Eq. (S2).
		(S3)

n and t* are determined by fitting logt to  linearly. 
S2 Activation energy calculation for the main thermal degradation stage of aerogels 
The Broido equation [2] can be used to determine the activation energy (Ea) of the main thermal degradation stage of the material. In the following equation:
	$ln(ln(1/y))=-E_{a}/RT+ln(RZ{T_{m}^{2}/E_{a}\beta)}$	(S4)

Here, y represents the undecomposed sample mass fraction; Tm is the temperature at the maximum thermal degradation rate (K); β denotes the heating rate (K/min); Ea is the activation energy (kJ/mol); R is the gas constant (8.314 J/(K·mol)). We can obtain Ea by calculating the slopes of $ln(ln(1/y))$-1/T linears.
S3 Cooling performance analysis of the aerogel samples
According to the previous research [3], under the irradiation of PF400 xenon lamp (China ANTOINE Company), the cooling simulation test of aerogel samples was carried out in the indoor environment at the irradiation power of 1.5 W/cm2 (the solar radiation intensity is about 1200 W/m2). To simulate sunlight, the sample was placed 25 cm below a xenon lamp. Infrared images and real-time temperatures were obtained with a Hikvision H21 Pro thermal imaging camera (Hikvision Company, China) (error: ±1.0°C). 
The outdoor solar radiation cooling experiment referred to the previous research [3]. The experiment was conducted on July 23, 2024 (summer solstice period) under clear sky conditions on the flat rooftop of a nine-story building at the University of Science and Technology of China (Hefei, China; longitude: 117.2550177; latitude: 31.8531187). Each incubator consists of a 20×20×20 cm3 insulated foam box lined with reflective foil. A transparent low-density polyethylene film seals the opening, serving as a wind barrier. Aerogel samples (15×15×2 cm3) are placed in grooves atop foam boxes positioned 1.5 m above ground level to minimize thermal conduction from the surface. A temperature sensor mounted directly on the specimen's rear surface monitors real-time thermal changes, with data continuously recorded by a K-type thermocouple (±0.1 °C uncertainty). For control measurements, an identical sensor array monitors ambient temperature at equivalent height. 
S4 Filtration test of the aerogel samples
The smoke filtration test was conducted in a custom-designed test chamber that has been reported in our previous research [4]. Smoke was generated by igniting smoke cakes in the combustion chamber of the smoke generation system, and subsequently delivered to the smoke collection chamber at a constant flow rate of 5 L/min. Aerogel samples were positioned at the inlet of the smoke collection chamber (Figure S35, 0.8×0.8×0.7 m3) for smoke filtration. The aerogel samples (5×5×1 cm3 and 5×5×0.5 cm3) were secured in a custom-fabricated mold allowing gas penetration. An electronic laser (520 nm, 2 W) paired with a laser receiver was installed in the smoke collection chamber. Visibility changes within the chamber were evaluated through continuous monitoring of laser intensity (I/It=0) variations detected by the receiver. Commercial activated carbon filter masks (3M Company) were used for comparison.
S5 The test for studying the effect of humidity and heat treatment time on aerogel stability
The aerogel samples were placed in a constant temperature and humidity chamber (STIK CTHI-150B, USA; Figure S28A) at 25 °C with relative humidity (RH) levels of 15%, 30%, 50%, 70%, 80%, 85%, and 90%. For thermal aging tests, aerogels were exposed to 70°C and 30% RH in the STIK environmental chamber for 4–12 days. The samples were then subjected to compressive tests, LOI tests and thermal conductivity tests. 


Figure S1 (A) Dynamic surface tension of the liquids for making aerogels at different lifetimes. (B) The fitted dynamic surface tension curve to determine adsorption kinetics parameters using Rosen's approach. (C) Adsorption characteristic time t* of the liquids for making aerogels. 
Note: Lower adsorption characteristic time means that molecules can adsorb to the gas-liquid interface faster during the foaming process. Formulations incorporating APG demonstrate the enhancement in adsorption kinetics.


Figure S2 Rheology of different foam templates under different ERs. 
Note: Increasing ER can improve the elasticity of foam. The addition of K-formate significantly improves the elasticity of foam by inducing the crystallization domain which was reported in our previous work [5]. The increase in the elastic modulus of foam is also due to the blockage of gelatin and PVA chains at the Plateau border.

Figure S3 Tensile strength of aerogels with or without K-formate. The network of linked gelatin and PVA chains in the aerogel functioned as a “scaffold” to propagate mechanical stress between the hydrophobic region and the binding chain. K-formate contributed to the hydrophobic interactions  (see Figure 1I) in the physically cross-linked region which acted as a “load carrier”, rapidly absorbing energy and sustaining significant deformation during aerogel stretching, which led to the high ductility and strength of the aerogel. 


Table S1 Screening of gelatin-based aerogel.
Polymer	Concentration (%)	Elasticity	Integrity
Polyurethane	2	++	++++
Polyurethane	4	+	++++
PVA	2	+++	++++
PVA	4	++++	++++
Cellulose nanofibers	1	++	++++
Cellulose nanofibers	2	+	++++
Cellulose nanofibers	4	+	++++
Cellulose nanocrystals	1	+	++++
Cellulose nanocrystals	2	+	++++
Cellulose nanocrystals	4	+	++++
Hydroxypropyl cellulose	2	++	++++
Hydroxypropyl cellulose	4	+	++++
Methyl cellulose	2	+	++++
Methyl cellulose	4	+	++++
Xanthan gum, arabic gum, guar gum	2	++	+++
Collagen	2	+	++

Note: Elasticity testing employs a qualitative assessment method utilizing manual finger pressure application to evaluate material deformation resistance and self-recovery potential. This technique measures both the degree of structural collapse under stress and the material's capacity for rapid shape restoration. Integrity assessment is conducted through visual inspection. The rating system utilizes a four-tier scale, where "+" indicates minimal elasticity or minimal integrity and "++++" represents optimal performance characteristics. The concentration of Ge is 5% and the concentration of APG is 1%.


Figure S4 Bubble coarsening process of the foam at ER=6.


Figure S5 The variation of bubble size distribution over time.


Figure S6 Morphology of ice templates and aerogels with and without APG. The upper figure shows the ice templates, and the lower figure shows the freeze-dried aerogels.


Figure S7 Foaming ability of the liquids determined by the standard Ross-Miles method [6].


Figure S8 Morphology of the foam templates at different times. High stability is conducive to maintaining the bubble structure of foam before the ice template is completely established.


Figure S9 Evolution of foam height with time. The foam made from Ge/PVA/APG shows the best stability.


Figure S10 SEM image of aerogel made from PVA/APG/K-formate without gelatine at ER=6. No dome structure and hierarchical structure can be observed in the PVA/APG/K-formate aerogel without gelatin.


Figure S11 Compressive resilience of the aerogels with and without gelatine. The weight has a mass of 1.0 kg.


Figure S12 Microscopic image of the aerogel with ER=0. The sample is made from Ge/PVA/APG/K-formate.


Figure S13 Mass of the aerogel at different ERs. Increasing ER can significantly reduce the density of aerogels. The sample is made from Ge/PVA/APG/K-formate.

Figure S14 Effect of the expansion ratio on compression performance of the Ge/PVA/APG (Ge:PVA=1:2) aerogels.
Note: The elastic behavior of the aerogel originates from the jammed state of bubbles in the foam template. At ER≤ER(bubble jamming transition in 3d foam), the bubbles in the template are spherical and do not touch, and the as-obtained aerogel is stiff. At ER(bubble jamming transition in 3D foam)<ER≤8, the bubbles touch and take the shape of a squashed sphere at each bubble/bubble contact, and the as-obtained aerogel is superelastic. At 8<ER, many bubbles in the template are spherical and do not touch with increasing ERs, and the as-obtained aerogel is too soft and loses its elasticity.


Figure S15 Bubble morphology of foam made from the compressed air foam generation system with different liquid fractions. The liquid fraction φ is the reciprocal of ER. The scale bar is 50 μm.
Note: As ER increases, the separated spherical bubbles gradually become jammed spherical bubbles, and eventually become polygonal bubbles. For the Tessari method, the ER can be varied independently of the bubble size distribution for the given formulation.


Figure S16 (a) Commercial compressed air foam generation system used for large-scale production of aerogel. The equipment is obtained from ISAFETY TECHNOLOGY CO., LTD.(http://www.ustc-safety.com). (b) Foam formation mechanism in the commercial compressed air foam generation system. 


Figure S17 Initial bubble size of foam produced by compressed-air foam system and Tessari method. The sample is made from Ge/PVA/APG/K-formate. 
Note: The Tessari method is difficult to manufacture bubble templates with a ER greater than 10. However, for the same liquid samples, the bubble size distribution of the bubble template generated by the Tessari method does not change with the change of ER. This is related to the difference in bubble formation mechanisms between the two methods. The Tessari method is convenient for us to study the effect of ER on the performance of aerogels under constant bubble size.


Figure S18 Thermal conductivity of aerogels (ER=6) at different ratio of Ge and PVA. The sample is made from Ge/PVA/APG/K-formate.


Figure S19 Thermal conductivity of the aerogels (ER=6) at different temperatures. The sample is made from Ge/PVA/APG/K-formate. At low temperatures, the aerogels have low thermal conductivity.

Table S2 Raw material cost of the superelastic aerogel in this work.
Component	Raw material cost (dollar/kg)	Reference
Gelatin	8.0	https://www.alibaba.com/product-detail/200-250-Bloom-Bovine-Gelatin-with_1601018066925.html?spm=a2700.galleryofferlist.wending_right.i4.2a6413a0ZsPxT2
PVA	2.9	https://www.alibaba.com/product-detail/Polyvinyl-Alcohol-PVA-2488-powder-Factory_1600829975103.html?spm=a2700.galleryofferlist.topad_classic.d_title.404413a05W3HHQ
APG	8.0	https://www.alibaba.com/product-detail/Yhealth-Alkyl-Glycoside-APG-0810-0814_1600947328524.html?spm=a2700.galleryofferlist.normal_offer.d_title.c99913a06bV8up
K-formate	1.5	https://www.alibaba.com/product-detail/Industrial-Grade-Potassium-Carbonate-De-Potassium_1600862348384.html?spm=a2700.galleryofferlist.normal_offer.d_title.14d513a06mgWHM
Raw material cost of the superelastic aerogel in this work (5.248 dollar/kg)


Table S3 Comparison of raw material cost, density and thermal conductivity of aerogel between this work and previous work.
Aerogel samples	Density (mg/cm3)	Thermal conductivity (mW/(m·℃))	Raw material cost (dollar/kg)	Reference
PVA/Ge/APG/K-formate (ER=6)	17	24	5.248	This work
CNF@PU	4.1	24	22	[8]
CNF/BA/CA	39.8	30	65	[23]
CNF foam	9.2	17.8	70	[25]
SCQs	260	17.4	50	[27]
CNF/GO	7.5	15	28	[26]
BC-Si3	90	15.3	130	[20]
c-CNF@PU	20.1	45.5	22	[8]
BGHA	150	39	115	[21]
BC-PMSQ	0.7	28.9	80	[22]
CNF-ANFA	5.9	17	46	[24]
Silica Aerogel #2	180	21.5	6.75	[28]
Silica Aerogel #1	24	34	4.8	[28]
PVA/Ge/APG/K-formate (ER=8)	12	21	5.248	This work
PVA/Ge/APG/K-formate (ER=0)	85	81	5.248	This work

Note: The cost analysis of aerogels is broken down into five different areas: materials, energy, manpower, facility costs, and equipment. The raw material costs dominate in all but a few cases. We selected low-cost Ge and PVA, which are intended to replace the expensive cellulose nanofibers (CNFs, its cost exceeds 1500 dollar/kg [8]). The production costs of freeze-dried aerogels generally involve the analysis of SWOT matrix. Weaknesses, strengths, threats and the most relevant opportunities must be considered [7,8]. The SWOT matrix identifies the substantial capital investment needed for core equipment (lyophilizer) as a notable threat, while highlighting the equipment's automated operation as a competitive strength. It should be emphasized that complete production cost analysis requires a comprehensive understanding of current market conditions and future projections. Given the unavailability of pertinent market data, this research cannot provide the complete production cost for Ge/PVA aerogel. Consequently, the present work is confined to raw material cost analysis.
Whether using freeze-drying, supercritical drying, or ambient pressure drying methods, raw material costs consistently dominate the composition of aerogel production [7,8]. Previous research on freeze-dried CNF aerogels demonstrates that raw materials account for approximately 70% of the production costs [8] (encompassing raw materials, device, utilities, and labor). Moreover, prolonging production duration enables effective amortization of the costs of the freeze-drying equipment. Previous research analyzing cash flow and timelines for CNF-reinforced polymer aerogel production via freeze-drying (the raw material cost of CNF-based aerogels exceeds 50 dollar/kg) demonstrated that positive cash flow emerges starting from the second production year, exhibiting an annual increasing trend [8]. Given that the raw material costs in our study (5.248 dollar/kg) are significantly lower than CNF, relatively lower production benefits are anticipated.


Figure S20 Structure of the aerogel sample with ER=3 after 100 cycles of compression. The sample is made from Ge/PVA/APG/K-formate.

Figure S21 Compressive stress-strain curve of aerogel at 40% strain under different Ge and PVA ratios. The sample is made from Ge/PVA/APG/K-formate.


Figure S22 SEM images of pore architecture evolution in Ge/PVA/APG/K-formate aerogel (ER=6) before and after 100 compression cycles. (A) Ge:PVA=1:0. (B) Ge:PVA=0:1. 


Figure S23 FTIR spectra for Ge, PVA and Ge/PVA
Note: When the cutting surface is wetted with deionized water, it swells into a hydrated film due to the hydrophilicity of Ge and PVA networks. In the hydrated film, both gelatin and PVA molecules can freely move. When the dry surface and wetted surface contact, the exposed Ge and PVA chains on the aerogel surface link through hydrogen bonding interactions, leading to the reconstruction of the aerogel network. Furthermore, since the hydrated film on one fracture surface is extremely thin while the other fracture surface is dry, the dry fracture surface can rapidly adsorb water molecules from the hydrated film, leading to an expanded surface area for water evaporation. Therefore, the contact region can dry quickly and form a denser layer. Consequently, the aerogel displays rapid healing capability. FTIR spectra prove this hydrogen bonding interaction (Figure S23). First, with the PVA addition, the O-H stretching absorption peak at 3324 cm-1 shifts to lower wavenumbers. This shift indicates hydrogen bonding interactions between PVA and Ge. The intensity of the peaks at 1654 cm-1 and 1549 cm-1 involving C=O stretching vibrations and C-N bond stretching vibrations increase. This suggests that the addition of PVA alters the conformation of Ge. The absorption peak at 1450 cm⁻¹ attributed to CH₂-OH bending vibration, more specifically O-H stretching, further shifts to lower wavenumbers with increasing PVA content. These changes demonstrate that PVA addition promotes stronger hydrogen bonding interactions between molecules in the system.  


Figure S24 Thermal conductivity and tensile strength of Ge/PVA/APG aerogels (ER=6) before and after recycling. The green column represents tensile strength, and the red column represents thermal conductivity.


Figure S25 (A) LOI of Ge/PVA/APG aerogels (ER=6) before and after multi-cycles. (B) LOI of Ge/PVA/APG aerogels (ER=6) before and after prolonged thermal aging.
Note: Recycled aerogels were regenerated via iterative cycles of heating, cooling, secondary foaming, and freeze-drying. For thermal aging tests, aerogels were exposed to 70°C and 30% relative humidity in the STIK environmental chamber for 4–12 days prior to LOI characterization. Notably, after four recycling iterations, the LOI values of the recycled aerogels showed low deviation (<5%) from those of the pristine samples, confirming the preservation of flame-retardant properties. Furthermore, aerogels retained exceptional flame retardancy even after 12 days of thermal aging at 70°C (Figure S25B). These findings collectively validate the material's ability to maintain stable flame-retardant performance through repeated recycling and prolonged thermal exposure.


Figure S26 Comparison of the FTIR spectra before and after degradation of the Ge/PVA/APG aerogels (ER=6) placed in soil.


Figure S27 Change of the density of Ge/PVA/APG aerogels (ER=6) with time in non-degradative indoor environments (Hefei, annual average RH:68%).

Figure S28 (A) Constant temperature and humidity chamber. (B, C) Compressive stress-strain curve of aerogel at 40% strain under different RH. (D) Thermal conductivity of aerogels under different RH. (E) Thermal conductivity of aerogels at different times under constant humidity (RH=90%). (F, G) SEM images of aerogels with and without PDMS modification.
Note: the aerogel samples were conditioned in a constant temperature and humidity chamber (STIK CTHI-150B, USA; Figure S28A) at 25 °C with relative humidity (RH) levels of 15%, 30%, 50%, 70%, 80%, 85%, and 90%. Following a 48-hour equilibration period (unless specified otherwise), the samples were subjected to compressive and thermal conductivity tests. Figure S28 summarizes the compressive behavior, thermal conductivity, and pore structure evolution of the aerogels under varying humidity. Below 80% RH, humidity had negligible effects on the compressive strength and thermal conductivity of Ge/PVA/APG aerogels (Figure S28B). However, at RH≥85% (after 48 hours), the aerogels displayed structural softening, skeleton adhesion (Figure S28G), and a decline in compressive stress (Figure S28B). The increased thermal conductivity observed at RH≥85% (Figure S28D) arises from the replacement of air (low thermal conductivity) with water (high thermal conductivity) within the pores. Remarkably, applying a commercial hydrophobic PDMS coating significantly enhanced moisture resistance. Even at 90% RH, the modified aerogels maintained robust elasticity (Figure S28C), low thermal conductivity (Figure S28D), and structural integrity of pores (Figure S28F). Long-term testing under 90% RH confirmed that the PDMS-coated aerogels retained low thermal conductivity after 96 hours of exposure (Figure S28E), demonstrating exceptional humidity stability.﻿


Figure S29 Calculation of activation energy of the aerogel samples during pyrolysis.


Figure S30 Raman spectra of char from (A) Ge/PVA/APG aerogels without adding K-formate and (B) Ge/PVA/APG aerogels with adding 1% K-formate. The ratio of peak D to peak G is usually used to evaluate the degree of graphitization of residual carbon. A lower ratio of peak D to peak G indicates an increase in the degree of graphitization after adding K-formate.


Figure S31 Reflectance spectra of Ge/PVA/APG/K-formate aerogels (ER=6) using UV-VIS-NIR devices. 


Figure S32 Changes in the temperature of the Ge/PVA/APG/K-formate aerogel samples under solar irradiation.
Note: For the aerogel with ER=6, we observed that the average cooling effect was about 3.2 °C during the cloudy period, while in the sunny period, the cooling effect soared to 12.5 °C.


Figure S33 Changes in the temperature of the Ge/PVA/APG/K-formate aerogel samples under xenon lamp.
Note: The aerogel with ER=6 achieves a cooling effect of 10.3 ℃ lower than the ambient temperature, which is mainly due to its super high solar reflectivity, which enables the aerogel to maintain effective radiation cooling.

Figure S34 Water resistance and contact angles of the Ge/PVA/APG aerogels (ER=6) modified with PDMS.


Figure S35 Setup for filtration test of aerogel samples.


Figure S36 The variation of normalized laser intensity over time for qualitatively describing the smoke density in the chamber. The ratio of light intensity to initial light intensity also indicates the visibility in the smoke collection compartment. A lower ratio indicates lower visibility, which is caused by smoke diffusion. 


Figure S37 Filtration pressure drop of the Ge/PVA/APG/K-formate aerogel used to filter the smoke.
Note: Compared with the commercial activated carbon mask filter, the aerogel in this study shows a low pressure drop (<70Pa) and high filtration efficiency. 


Reference
Hua X., Rosen M., Dynamic surface tension of aqueous surfactant solutions, Journal of Colloid and Interface Science, 124 (1988) 652-659.
Broido A. A simple, sensitive graphical method of treating thermogravimetric analysis data, Journal of Polymer Science Part A-2: Polymer Physics, 7 (1969) 1761-1773.
Cai W., Lin B., Qi Li., et al., Bio-based and fireproof radiative cooling aerogel film: Achieving higher sustainability and safety, Chemical Engineering Journal, 488 (2024) 150784.
Li Q., Lin J., Li C., Lu S., Chen X., Effect of polyvinyl chloride fire smoke on the long-term corrosion kinetics and surface microstructure of tin–lead and lead-free solders, Journal of Materials Science: Materials in Electronics, 30 (2019)16395-16406.
Yu X., Li H., Qiu K., et al., One-step construction of light-responsive intelligent foam by the Hoffmeister effect and 2D black phosphorus: High stability and on-demand degradation, Journal of Materials Science & Technology, 211 (2025) 98-109.
Jiang N., Sheng Y., Li C., Lu S., Surface activity, foam properties and aggregation behavior of mixtures of short-chain fluorocarbon and hydrocarbon surfactants, Journal of Molecular Liquids, 268 (2018) 249-255.
[7] Simón-Herrero C., Romero A., Dorado F., Gracia I., Valverde J., Sanchez-Silva L., Taylor-made aerogels through a freeze-drying process: economic assessment, Journal of Sol-Gel Science and Technology, 89 (2019) 436-447.
[8] Víctor-Román S., Simón-Herrero C., Romero A., Gracia I., Valverde J., Sánchez-Silva L., CNF-reinforced polymer aerogels: Influence of the synthesis variables and economic evaluation,  Chemical Engineering Journal, 262 (2015) 691-701.
